# TET Enzymes and 5hmC Levels in Carcinogenesis and Progression of Breast Cancer: Potential Therapeutic Targets

**DOI:** 10.3390/ijms25010272

**Published:** 2023-12-24

**Authors:** Eric Genaro Salmerón-Bárcenas, Ana Elvira Zacapala-Gómez, Francisco Israel Torres-Rojas, Verónica Antonio-Véjar, Pedro Antonio Ávila-López, Christian Johana Baños-Hernández, Hober Nelson Núñez-Martínez, Roberto Dircio-Maldonado, Dinorah Nashely Martínez-Carrillo, Julio Ortiz-Ortiz, Hilda Jiménez-Wences

**Affiliations:** 1 Departamento de Biomedicina Molecular, Centro de Investigación y de Estudios Avanzados del Instituto Politécnico Nacional, Ciudad de México C.P. 07360, Mexico; eric.salmeron@cinvestav.mx (E.G.S.-B.); pedroavila@cinvestav.mx (P.A.Á.-L.); 2 Laboratorio de Biomedicina Molecular, Facultad de Ciencias Químico-Biológicas, Universidad Autónoma de Guerrero, Chilpancingo C. P. 39090, Guerrero, Mexico; 17757@uagro.mx (A.E.Z.-G.); ftorres@uagro.mx (F.I.T.-R.); 11335@uagro.mx (V.A.-V.); julioortiz@uagro.mx (J.O.-O.); 3 Instituto de Investigación en Ciencias Biomédicas, Centro Universitario de Ciencias de la Salud, Universidad de Guadalajara, Guadalajara C. P. 44340, Jalisco, Mexico; johana.banos@academicos.udg.mx; 4 Departamento de Genética Molecular, Instituto de Fisiología Celular, Universidad Nacional Autónoma de México, Ciudad de México C. P. 04510, Mexico; hnunez@ifc.unam.mx; 5 Laboratorio de Investigación Clínica, Facultad de Ciencias Químico-Biológicas, Universidad Autónoma de Guerrero, Chilpancingo C. P. 39090, Guerrero, Mexico; rdircio@uagro.mx (R.D.-M.); dnmartinez@uagro.mx (D.N.M.-C.); 6 Laboratorio de Investigación en Biomoléculas, Facultad de Ciencias Químico-Biológicas, Universidad Autónoma de Guerrero, Chilpancingo C. P. 39090, Guerrero, Mexico

**Keywords:** breast cancer, TET1, TET2, TET3, 5hmC

## Abstract

Breast Cancer (BC) was the most common female cancer in incidence and mortality worldwide in 2020. Similarly, BC was the top female cancer in the USA in 2022. Risk factors include earlier age at menarche, oral contraceptive use, hormone replacement therapy, high body mass index, and mutations in *BRCA*1/2 genes, among others. BC is classified into Luminal A, Luminal B, HER2-like, and Basal-like subtypes. These BC subtypes present differences in gene expression signatures, which can impact clinical behavior, treatment response, aggressiveness, metastasis, and survival of patients. Therefore, it is necessary to understand the epigenetic molecular mechanism of transcriptional regulation in BC, such as DNA demethylation. Ten-Eleven Translocation (TET) enzymes catalyze the oxidation of 5-methylcytosine (5mC) to 5-hydroxymethylcytosine (5hmC) on DNA, which in turn inhibits or promotes the gene expression. Interestingly, the expression of TET enzymes as well as the levels of the 5hmC epigenetic mark are altered in several types of human cancers, including BC. Several studies have demonstrated that TET enzymes and 5hmC play a key role in the regulation of gene expression in BC, directly (dependent or independent of DNA de-methylation) or indirectly (via interaction with other proteins such as transcription factors). In this review, we describe our recent understanding of the regulatory and physiological function of the TET enzymes, as well as their potential role as biomarkers in BC biology.

## 1. Introduction

Among female cancers, Breast Cancer (BC) is the most commonly diagnosed cancer, with 2,026,419 new cases and 684,996 new deaths estimated worldwide in 2020. In countries with very high or high Human Development Index (HDI), BC is the most frequent cancer with a very HDI high/high [1]. In the United States, a total of 287,850 new cases and 43,250 new deaths were estimated in 2022 [2]. The main risk factors for BC include earlier age at menarche, later age at menopause, nulliparity, low parity, delayed age at first birth, oral contraceptive use and hormone replacement therapy, rising body mass index, mutations in *BReast CAncer gene (BRCA)* 1/2 genes, and a diet rich in fat and animal-source products [3,4,5,6,7]. BC can be classified into four subtypes based on gene expression profiles, including Luminal A, Luminal B, Erb-B2 Receptor Tyrosine Kinase 2 (HER2)-like, and Basal-like. The Luminal A subtype is characterized by Estrogen Receptor (ER+) and/or Progesterone Receptor (PR+) expression. The Luminal B subtype also expresses ER+/PR+, however, it correlates with worse prognosis. The HER2-like subtype is characterized by the amplification of the *HER2* receptor gene. The Basal-like subtype, also known as Triple-Negative Breast Cancer (TNBC) does not express ER, PR, and HER2 receptors (ER-, PR-, and HER2-) [8]. Chemical modifications of DNA have been one of the best-characterized epigenetic marks in BC, particularly DNA methylation and 5-methylcytosine (5mC) [9,10]. However, the role of Ten-Eleven Translocation (TET) enzymes and 5-hydroxymethylcytosine (5hmC) in BC is less understood. In this work, we describe our recent understanding of the regulatory and physiological function of the TET enzymes, as well as their potential role as biomarkers in BC biology.

## 2. Discovery of TET Enzymes and 5hmC

**Figure 1:**
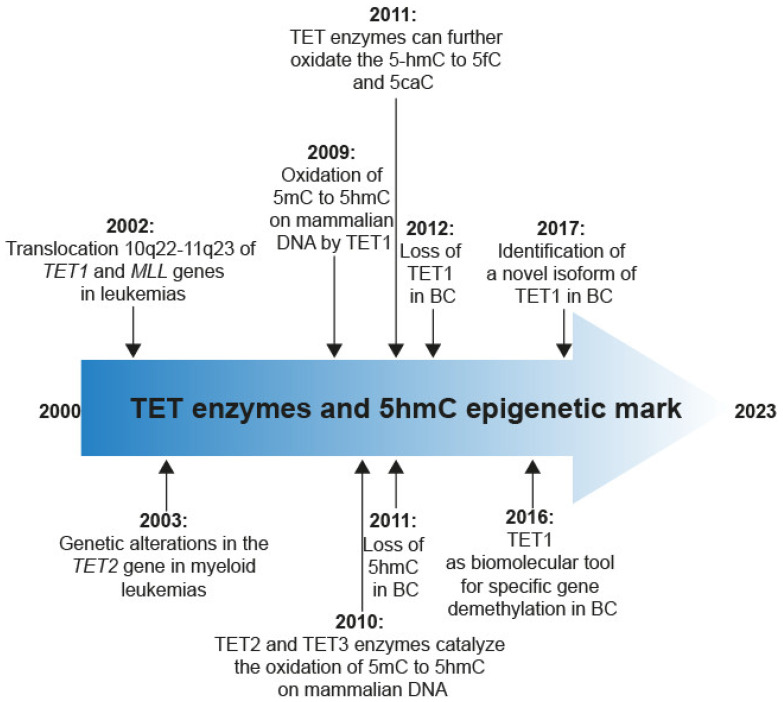
Timeline of main findings of TET enzymes.

The TET family proteins consist of three dioxygenase enzymes: TET1, TET2, and TET3. Computational phylogenetic studies have revealed homology between *TET1*, *TET2,* and *TET3* genes [11]. As shown in Figure 1, in 2002, a genetic alteration in the *TET1* gene was identified through the detection of a chromosomal rearrangement, specifically the translocation 10q22-11q23 of *TET1* and *Lysine Methyltransferase 2A (MLL1)* genes in acute myeloid leukemias [12,13]. Next year, in 2003, several deletions and mutations were identified in the *TET2* gene in myeloid leukemias [14]. Interestingly, in 2009, it was first reported that the TET1 enzyme catalyzes the oxidation of 5mC to 5hmC in mammalian DNA in a 2-oxo-glutarate and Fe (II)-dependent manner [11,15]. Subsequently, it was reported that the TET2 and TET3 enzymes also catalyze the 5mC oxidation to 5hmC [16]. In 2011, it was reported that TET enzymes can further oxidate the 5hmC to 5-formylcytosine (5fC) and 5-carboxylcytosine (5caC) [17]; these last two modifications are recognized and replaced by an unmodified cytosine through the Base Excision Repair (BER) pathway, leading to transcriptional activation induced by TET enzymes and 5hmC [18]. At the same time, it was reported that the 5hmC levels are decreased in BC [19]. One year later, a low expression of TET1 enzyme in BC was shown [20]. Finally, TET1 was used for promoter-specific demethylation in a BC cell line in 2016.

## 3. Mechanism of TET-Mediated 5hmC

**Figure 2:**
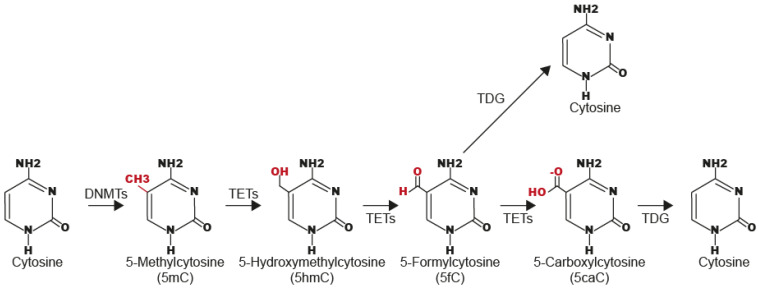
Mechanism of active demethylation of DNA by TET enzymes.

Currently, it is well known that TET enzymes participate in two processes of DNA demethylation, called active (or direct) and passive (indirect) demethylation. In the first process, TET enzymes oxidate the 5mC to 5hmC [11,16,21], which is transformed to 5-formylcytosine (5fC), and 5-carboxylcytosine (5caC) [17] (Figure 2). The 5fC and 5caC are removed by Thymine-DNA Glycosylase (TDG) enzyme and replaced by unmodified cytosines via the Base-Excision Repair (BER) pathway [22,23,24] (Figure 2).

In the second process (also called replication-dependent passive demethylation), TET enzymes oxidates the 5mC to 5hmC, which is not well recognized by Ubiquitin-Like PHD And RING Finger Domain-Containing Protein 1/DNA Methyltransferase 1 (UHRF1/DNMT1) complex, which participates in the maintenance of methylation patterns on DNA; therefore, the DNA methylation is decreased passively across each cell division [25].

Therefore, TET enzymes play a key role in DNA demethylation (passive or active) and their misregulation affects cancer-related genes, such as migration, invasion, apoptosis-related genes.

## 4. Expression of *TET* Genes and 5hmC Levels in BC

TET1 expression is decreased in BC tissue samples compared to normal breast tissue samples and correlates with larger tumors, advanced stage, lymph node status, and poor Overall Survival (OS) [26,27,28,29]. In addition, TET1 nuclear localization is decreased in invasive and in situ ductal BC tissue samples compared to normal breast tissue samples and correlates with low 5hmC levels [30]. Interestingly, between subtypes of BC, TET1 expression is only decreased in Luminal A, Luminal B, and HER2 subtypes of BC tissue samples compared to normal breast tissue samples [31], suggesting that TET1 could have a specific subtype-related role. Conversely, two studies reported that TET1 expression is increased in TNBC tissue samples compared to normal breast tissue samples, and its high expression correlates with poor OS and obesity in TNBC patients [32,33]. These contradictory results could be attributed in part to the study population, given that the first studies analyzed TET1 expression in several study populations [26,27,28,29], while the second studies used the same study population, specifically the TCGA dataset [32,33]. Moreover, TET1 expression positively correlates with 5hmC levels and negatively correlates with 5mC levels in TNBC patients [31].

TET1 expression is decreased in lymph node metastasis BC tissue samples and metastatic BC cells (YCC-B1, MDA-MB-231, and BT549) compared with normal breast tissue samples and non-metastatic BC cells (BT474 and SK-BR3), and its expression inversely correlates with methylation level in its promoter [34,35]. Conversely, a study reported that TET1 expression is increased in Lymph Node Metastasis BC tissue samples compared with metastasis-negative tissue samples [36]. These inconsistent results could be explained by the type of analyzed molecule and the technique used in each study; the first studies analyzed the TET1 expression at mRNA level by real-time PCR assays [34,35], while in the second study, TET1 expression was analyzed at the protein level by IHC assays [36].

Recently, a novel TET1 isoform known as TET1^ALT^ has been identified, and its expression is increased in BC tissue samples and cells (HCC2218, HCC1599, MCF7, MDA-MB-231) compared with adjacent normal breast tissue samples and untransformed immortalized cells (HMLE and MCF10A). The TET1^ALT^ isoform expression correlates with poor OS and degree of differentiation of tumor in BC patients [37,38]. Interestingly, the expression of this isoform is predominant in TNBC tissue samples [32,33].

TET2 expression is decreased in metastatic, TNBC, ER+/PR+ BC, and BC tissue samples compared to Luminal subtype BC and normal breast tissue samples. Its low expression correlates with poor OS, Relapse-Free Survival (RFS), Post-Progression Survival (PPS), advanced stage, lymph node metastasis, high tumor grade, and aberrant methylation in its promoter in BC patients [28,33,39,40,41,42]. Low TET2 expression negatively correlates with the histological classification of BC lesions, activated CD8^+^ T cells, CD56^+^ natural killer cells, gamma delta T cells, macrophages, myeloid-derived suppressor cells (MDSC), and monocytes and positively correlates with 5hmC levels, immune-infiltrating tumor-associated fibroblast, and memory B cells [41,43]. In contrast, other studies showed that TET2 expression is increased in lymph node metastasis, BC tissue samples, and MCF-7 BC cells compared with metastasis-negative, normal tissue samples and HBL-100 untransformed immortalized cells [36,44,45]. These incongruent results could be due to key factors for each study, including a small sample size [28], technique used (such as RNA-seq) [33], analysis method (2^−∆∆ct^) [44,45], and the analyzed molecular level (at protein level by IHC) [39]. Moreover, TET2 expression decreases in the SUM149 TNBC cell line, metabolically adapted cells, cultivated in glutamine-free medium, suggesting that cells adapted have an epigenetic state that does not require high TET2 levels [46]. Interestingly, TET2 nuclear localization is decreased in invasive and in situ ductal BC tissue samples compared to normal breast tissue samples and correlates with higher BMI, lymph node metastasis, poor differentiation, and basal-like molecular subtype [30].

TET3 expression is decreased in BC tissue samples and MFC-7 BC cells compared with normal breast tissue samples and HBL-100 BC cells [28,44], while its expression is increased in TNBC, HR+, and HER+ subtypes and BC tissue samples compared with normal breast tissue samples [33,45]. The opposite results between these studies could be due to differences in the sample size [28,33] and analysis method (2^−∆∆ct^) [44,45]. Similarly, TET2 and TET3 expression are increased in peripheral blood mononuclear cells from BC patients compared with healthy donors, suggesting their involvement in the overexpression of immune checkpoint and ligand genes through demethylation of their promoters [47]. On the other hand, a six-genes panel, including TET3, was identified as a practical prediction model for the pathological complete response to neoadjuvant chemotherapy in patients with TNBC [48]. After surgery, high TET3 expression correlates with better Disease-Free Survival (DFS) in BC patients that received chemotherapy, including anthracyclines [26]. TET1, TET3, and 5hmC levels are associated with tumor hypoxia, advanced differentiation grades, poor OS, and DFS in BC patients [49].

The levels of 5hmC are decreased in metastatic, invasive, and in situ BC tissue samples compared with matched cancer or normal breast tissue samples [28,42,50], while the 5fC and 5caC levels are increased in BC tissue samples compared with tumor-adjacent tissue samples. A detailed analysis revealed that the levels of 5mC, 5hmC, 5fC, and 5caC could be used to distinguish between different BC subtypes [51]. Low 5hmC levels correlate with poor cell differentiation, Disease-Specific Survival (DSS), and DFS in BC patients, including the ER/PR negative subtypes [30]. Moreover, the levels of this epigenetic mark negatively correlate with the histological classification of BC lesions and positively correlate with TET2 expression [43]. The 5hmC levels are increased, while the 5mC levels are decreased in tumorspheres obtained from MCF-7 cells. In these tumor spheres, the TET3 expression is increased and could be involved in the Programmed Cell Death 1 Ligand 1 (PD-L1) over-expression, an immune checkpoint ligand, by demethylation of its promoter [52]. Finally, a study showed that the 5hmC levels could be determined using a novel electrochemical immunosensor in BC [53].

## 5. Genetic Alterations in *TET* Genes in BC

Some Single Nucleotide Polymorphisms (SNPs) and mutations associated with TET2 expression have been identified in BC patients. The Reference SNP(rs)62331150 and rs73838678 SNP are located at enhancer regions of the *TET2* gene, however, only the T allele of rs62331150 SNP is associated with low TET2 expression in BC tissue samples from women of European ancestry [54]. The rs9790517 SNP, c.1085_1086insT (insertion), c.2072delC (deletion), c.3646C>T, c.4361_4362insG (insertion), and c.3812_3820delGCGCCTGTC (deletion), loss-of-function variants (changes in coding region or CDS), as well as theirs changes at protein level (ID: p.Pro363SerfsTer6, p.Thr691MetfsTer9, p.Arg1216Ter, p.Arg1455GlnfsTer23, and p.Cys1271_Gln1274delinsTer) have been identified in the *TET2* gene in the familial breast cancer families group from the Australian population. Interestingly, the c.832C > T (CDS change) and c.1458delC (deletion) loss-of-function variants, and their changes at protein level (ID: p.Gln278Ter and p.Asn486LysfsTer11), were identified in the population-matched cancer-free group [55]. The microRNA-related SNP *TET2*-rs7670522(A>C) was identified in patients with BC, and the A allele is associated with an increased BC risk in Australian Caucasian populations [56].

A study identified deletions in the *TET1* gene and revealed that this gene was the most commonly deleted (8.6%) in metastatic TNBC tissue samples from The Metastatic Breast Cancer Project [57]. On the other hand, a high prevalence of insertion-deletion mutations in the *TET2* gene was identified in premenopausal Asian patients with HR-positive and HER-negative BC [58].

Deletion mutations and copy loss in the *TET2* gene have been identified in BC patients from the Taiwanese population and correlate with poor DFS [59]. The p.Q1702* (in a nonsense allele) and p.E1874K (change) somatic mutations were found in the *TET2* gene in tumor-infiltrating leukocytes from patients with primary TNBC [60], and the Q548* (substitution-nonsense) truncating mutation in the *TET2* gene was identified in a patient with solid papillary carcinoma with reverse polarity BC [61]. Somatic mutations, including nonsense and in-frame insertion (N1260S and E1151*) were found in the *TET2* gene in 2/6 patients with metaplastic BC [62]. The mutation R1516*/Q (missense or nonsense mutation) was identified in a patient with BC [41]. Finally, 10 mutations (ID: pTrh497Ser, p.Tyr819*, pHis1904Arg, p.Gln383*, p.Thr1069fs, p-Leu412_Pro413fs, p.Gly1370Val, p.Pro554_Pro555fs, p.Ser 385fs, and p.Gly429Arg) in TET2 protein were found in eight patients with therapy-related myeloid neoplasms following treatment for BC [63].

## 6. Molecular Mechanisms of TET Enzymes in BC

*TET1* promoter is methylated in T47D, MDA-MB-453, MCF-7, MDA-MB-231, and YCC9-B1 BC cells, and the treatment with 5-azacytidine (5-aza) and Trichostatin A (TSA) decreases the methylation in the *TET1* promoter, which promotes an increase in its expression in MDA-MB-231 BC cells [34,37,44,64].

Nuclear Factor Kappa B (NF-kB) transcription factor binds to the *TET1* gene promoter and inhibits its expression in MDA-MB-231, Hs578T, and BT-549 BC cells [31]. High Mobility Group AT-Hook 2 (HMGA2) is a non-histone architectural transcription factor that inhibits TET1 expression. HMGA2 down-regulation increases TET1 expression, which binds to its promoter and promotes its demethylation. TET1 re-expression induces Homeobox A9 (HOX9A) expression via demethylation of its promoter, decreasing cell invasion, tumor growth, and intravasation in MDA-MD-231 and MDA-MB-436 BC cells [65]. Enhancer Of Zeste 2 Polycomb Repressive Complex 2 Subunit (EZH2) is the catalytic subunit of Polycomb Repressive Complex 2 (PRC2) complex and inhibits TET1 expression through increasing H3K27me3 levels in MDA-MB-231, MDA-MB-436, and MDA-MB-453 TNBC cells. EZH2 down-regulation increases TET1 expression, which induces p53 expression through the demethylation of its promoter. p53 induces cellular senescence in the TNBC cells and in vivo [66]. In addition, TSA induces TET1 expression, which inhibits cell invasion by decreasing Matrix Metallo Proteinases (MMP) 2/9 expression via Tissue Inhibitor Of Metalloproteinases (TIMP) 2/3 re-expression in MCF-7 BC cells [67]. 

The miR-29a inhibits TET1 expression by targeting its 3′-untranslated region (UTR), leading Epithelial-to-Mesenchymal Transition (EMT), cell cycle progression, proliferation, and migration in MFC-10A, MCF-7, MDA-MB231, and MDA-MB-453 BC cells. However, TET1 re-expression reverses these processes [68]. miR-646 targets the TET1 3′-UTR and decreases its expression, promoting the down-regulation of Iroquois Homeobox 1 (IRX1) expression via methylation of its promoter in MCF-7 and MDA-MB-231 BC cells and in vivo. IRX1 down-regulation increases Histone H2B type 2-E (HIST2H2BE) expression, inhibiting apoptosis and promoting cell proliferation, migration, and invasion in these BC cells. However, TET1 re-expression down-regulates the effect of miR-646 on the progression of BC [29]. The miR-27a-3p down-regulates TET1 expression, which inhibits the Adenylyl Cyclase type 6 (ADCY6) expression by aberrant methylation in its promoter, promoting the cell proliferation, migration, and invasion in MCF-7 and MDA-MB-231 BC cells, while TET1 re-expression rescues these effects [69]. The miR-29b inhibits TET1 expression, while its re-expression decreases cell proliferation, invasion, and migration in MCF-7 and MDA-MB-231 BC cells [70]. FLI1 exonic circular RNA 1 (FECR1) is a circRNA that induces Friend Leukemia Virus Integration 1 (FLI1) expression to promote cell invasion in MDA-MB-231 BC cells. Mechanistically, FECR1 recruits TET1 to the *FLI1* promoter and demethylates it, increasing FLI1 expression [71]. Lastly, TET1 could increase Leucine Zipper Tumor Suppressor 1 (LZTS1) Tumor Suppressor Gene (TSG) expression through an increase in 5hmC levels in its promoter in BC patients [72].

The transcription factor Forkhead Box A1 (FOXA1) promotes local DNA demethylation through its interaction with TET1 and TET2 in vitro and in vivo in MCF-7 BC cells [73]. TET1 inhibits cell invasion and adhesion in BT474 BC cells [35], as well as the cell proliferation and tumor growth through the re-expression of *TIMP2* and *TIMP3* genes via demethylation of their promoters in MDA-MB-231, MCF-7, and MDA-MB-468 BC cells [27]. In contrast, TET1 promotes cell proliferation and migration through the activation of oncogenic signaling pathways, such as PI3K/AKT and mTOR, through the expression of genes involved in these signaling pathways via demethylation of their promoters in MDA-MB-231 and Hs578T TNBC cells [33]. These results may be masked by over-expression of TET1^ALT^ isoform, which is located mainly in cytoplasmic in MCF-7 BC cells and acts as an oncogene in vivo [38] by activating the PI3K pathway, promoting the cell proliferation and migration in Hs578T and MDA-MB-231 BC cells [33]. Additionally, Catalase (CAT) reprimes the TET1 expression, inhibiting the TARDBP expression by decreasing hydroxymethylation levels in its promoter in MDA-MB-468, MDA-MB-231, SUM149, and HCC70 BC cells, decreasing the formation of mammospheres and Cancer Stem Cells (CSCs) [32]. Lastly, TET1 induces MAP7 Domain-Containing 1 (MAP7D1) expression, promoting cell proliferation and invasion in MCF-7 cells [36]. 

TET2 expression is necessary to maintain the 5hmC levels in MCF-7 BC cells [74]. Estrogen Receptor 1 (ERα) induces TET2 expression through the recruitment of Myeloid/Lymphoid Or Mixed-Lineage Leukemia Protein 3 (MLL3) to an enhancer in the *TET2* gene in MCF-7 BC cells. TET2 decreases the methylation level in active enhancers and recruits ERα, increasing the expression of genes involved in response to hormone stimulus and cell proliferation [75]. Lysine Demethylase 2A (KDM2A) is a lysine demethylase that reduces TET2 expression through the interaction with the V-Rel Avian Reticuloendotheliosis Viral Oncogene Homolog A (RelA) transcription factor in MDA-MB-231, Hs578T, and MDA-MB-468 BC cells. However, TET2 re-expression increases Epithelial Cell Adhesion Molecule (EpCAM) expression via demethylation of its promoter, reducing the cell migration and invasion [39]. Finally, Estrogen (E2) deprivation leads to increased methylation in enhancers due to TET2 down-regulation in MCF-7 BC cells [76].

The miR-26b-5p and miR-29c-5p inhibit TET2 and TET3 expression, leading to a decrease in 5hmC levels in MCF-7 BC cells. However, the Lymphoid Specific Helicase (LSH) over-expression decreases miR-26b-5p and miR-29c-5p expression. LSH and TET2 form a complex, increasing the 5hmC levels in these cells [42]. The miR-22 decreases TET1, TET2, and TET3 expression by binding to their 3′-UTR regions. TETs’ down-regulation decreases 5hmC levels and increases 5mC levels in the miR-200a and miR-200c promoters, leading to an increase in Zinc Finger E-Box Binding Homeobox (Zeb)1, Zeb2, and B Lymphoma Mo-MLV Insertion Region 1 Homolog (Bmi1) expression, however, TET2 and TET3 re-expression rescue these processes [77]. In MCF-7 and MDA-MB-231 BC cells, miR-660-5p targets the TET2 3’UTR and down-regulates its expression. However, TET2 re-expression increases apoptosis and decreases the viability, migration, and invasion by down-regulation of PI3K/AKT/mTOR signaling in these same BC cells [78].

TET2 interacts with Forkhead Box P1 (FOXP1) to demethylate *Estrogen Receptor 1 (ESR1)*, *Trans-Acting T-Cell-Specific Transcription Factor GATA-(GATA3)*, and *FOXA1*, inducing their expression to promote mammary luminal lineage specification. Loss of TET2 expression favors the resistance to tamoxifen treatment in vivo [40]. Smad Nuclear Interacting Protein 1 (SNIP1) transcription factor recruits TET2 to regulate the V-Myc Avian Myelocytomatosis Viral Oncogene Homolog (c-MYC) target gene, promoting cell viability and DNA damage response in MCF-7 BC cells [79]. Interestingly, TET2 inhibits the PD-L1 expression independently of its catalytic activity, and TET2 recruits Histone Deacetylase (HDAC) 1/2 to PD-L1 promoter to deacetylate H3K27ac in MCF-7 and MDA-MB-231 BC cells [80].

Over-expression of TET2 Catalytic Domain (TET2-CD) increases the 5hmC levels, which induces and inhibits the expression of thousands of genes that inhibit cell proliferation and migration in MCF-7 BC cells, as well as tumor growth in vivo. Interestingly, TET2-CD alters the 5hmC in binding sites for the V-Myc Avian Myelocytomatosis Viral Oncogene Homolog (MYC) transcription factor in a set of genes involved in lysosome biogenesis [81]. On the other hand, TET2 knockout decreases the 5hmC levels and increases the 5mC levels, particularly in enhancers, altering the expression of genes, including ERα target genes, by reducing the ERα transcription factor binding. This promotes anchorage-independent and tumor growth in MCF-7 BC cells and in vivo [82]. Therefore, TET2 is indispensable to ER binding to target genes via GATA3 in MCF-7 BC cells [83].

## 7. Alterations of TET Enzymes in Hypoxic BC 

Several studies have shown that hypoxia promotes BC, particularly favoring a more aggressive phenotype, major risk of metastasis, and the probability of resistance to therapy [84,85]. Hypoxia reduces TETs’ activity and decreases 5hmC levels in MCF-10A and MCF-7 BC cells. Under hypoxic conditions, the 5hmC levels decrease mainly at promoters and enhancers, while 5mC levels increase, decreasing the expression of genes in MCF-7 BC cells. Particularly, there is an increase in 5mC levels and depletion in 5hmC levels in TSG, but not in oncogenes in BC tissue samples [74]. On the other hand, hypoxia increases TET1 and TET3 expression through the binding of the Hypoxia Inducible Factor 1 Subunit Alpha (HIF1α) transcription factor to their promoters in breast primary cultures and MCF-7 and MDA-MB-231BC cells. Then, TET1 and TET3 form a complex, which increases 5hmC levels in the *Tumor Necrosis Factor-alpha* (TNF*α*) gene promoter and induces its expression, activating the TNFα-p38-MAPK signaling pathway upon hypoxia conditions. However, TET1 and TET3 down-regulation sensitizes the breast tumor-initiating cells to paclitaxel and decreases the tumor growth and metastasis in vivo through down-regulation of the TNFα-p38-MAPK signaling pathway [49]. Similarly, TET3 increases the 5hmC levels in the binding site to the E2F1 transcription factor in the *Epithelial Splicing Regulatory Protein 1 (ESRP1)* promoter, inducing its expression under hypoxic conditions in MCF-7 BC cells [86]. Hypoxia attenuates the innate immune response in BC through miR-25/93 expression by increasing 5hmC levels within the miR-25/93 loci under hypoxia conditions in MCF-7 and MDA-MB-231 BC cells [87].

## 8. TET Enzymes as Potential Therapeutic Targets

Dimethyloxaloylglycine (DMOG) is a non-specific inhibitor of 2-OG-dependent dioxygenase that inhibits TET1, TET2, and TET3 expression, which increases the methylation in the *Major Histocompatibility Complex, Class I, G (HLA-G)* gene promoter and decreases its expression in MCF-7 BC cells [44]. 

Vitamin C is a co-factor of TET enzymes that increases 5hmC levels and promotes apoptosis mediated by Tumor Necrosis Factor Ligand Superfamily Member 10 (TRAIL) expression in MDA-MB-231 BC cells [88]. Conversely, vitamin C decreases the Mesenchymal-to-Epithelial Transition (MET), and apoptosis and increases cell proliferation and invasion through down-regulation expression in MDA-MB-231 BC cells with miR-302/363 cluster overexpression, which acts as tumor suppressor miRNAs [89]. 

3,6-Dihydroxyflavone (3,6-DHF) is a flavonoid component that inhibits BC by inhibiting DNMT1 activity and inducing the TET1 expression via demethylation of its promoter. Then, TET1 re-expression increases the miR-3a expression by demethylation of its promoter in MDA-MB-231 BC cells and in vivo [90]. 

Curcumin is an active component of the herb *Curcuma longa* that decreases cell proliferation in HCC-38, UACC-3199, and T47D BC cells. Curcumin increases TET1 expression by down-regulation of miR-29b and TET1 re-expression, which induces *BRCA1* TSG expression via demethylation of its promoter in HCC-38 BC cells. On the other hand, curcumin increases miR-29b expression and decreases TET1 expression, which decreases Synuclein Gamma (SNCG) oncogene expression via methylation of its promoter in T47D BC cells [91]. 

Di-(2-Ethylhexyl) phthalate (also known as DEHP), a synthetic plasticizer agent, promotes MCF-7 BC cell viability, in part by down-regulation of the TET1 expression [92]. 

All-trans retinoic acid promotes the interaction between Retinoic Acid Receptor Beta (RARβ) and TET2, as well as their nuclear localization in MCF12A, T47D, and MDA-MB-231 BC cells. The RARβ-TET2 complex induces miR-200c expression through the increases in 5hmC levels of its promoter in MCF12A BC cells. The miR-200c re-expression decreases Protein Kinase C Zeta (PRKCζ) expression, which decreases the CSC-like population in T47D and MDA-MB-231 BC cells [93]. 

Wild Yam Root Extracts (WYREs) from *D. villosa* are steroidal saponins and sapogenins, which inhibit the cell viability in MCF-7 and MDA-MB-231 BC cells. WYRE increases TET3 expression, while decreasing TET1 expression and 5hmC levels in MDA-MB-231 BC cells. Interestingly, WYRE increases GATA3 expression by demethylation of its promoter in MDA-MB-231 BC cells [94]. 

Dioscin (DS), an active component from WYRE, decreases cell proliferation, invasion, and migration in MCF-7 and MDA-MB-231 BC cells. DS decreases TET1 expression, while increasing TET2 and TET3 expression, increasing GATA3 expression by demethylation of its promoter in MDA-MB-231 BC cells. DS promotes MET through the activation of the GATA3 signaling pathway in MDA-MB-231 BC cells [95]. 

CAK-SKL is a recombinant antioxidant enzyme that deregulates the expression of genes and modified 5hmC levels, which positively correlate in MDA-MB-468 TNBC cells. CAK-SKL does not affect the TET1 expression, however, it modifies the distribution of 5hmC to be uniform across all genomic regions [96]. 

Hormone treatment with E2 and Gonadotropin Releasing Hormone (GnRH) decreases TET1 expression and increases the TET1^ALT^ isoform in T47D and MCF-7 BC cells [38]. Bisphenol A (BPA) and Bisphenol S (BPS) are endocrine disruptors that target estrogen receptors (ER) and induce cell proliferation in MCF-7, T47D, and MDA-MB-231 BC cells. DMOG increases cell proliferation in MCF-7 BC cells treated with BPA and BPS. BPA and BSP decrease TET2 expression and 5hmC levels in MCF-7 BC cells, at the same time inducing DNMT1 and DNA Methyltransferase 3 Alpha (DNMT3A) expression through ERα. DNMT1 and DNMT3A decrease 5hmC levels, moreover, they decrease TET2 expression by methylation of its promoter and promote cell proliferation in MCF-7 cells treated with BPA and BPS, however, TET2 over-expression partially rescues these results [97]. E2 treatment promotes the binding of ERα transcription factor to upstream enhancers of the *TET2* gene, increasing its expression as well as the 5hmC levels in MCF-7 BC cells [98]. Glyphosate, an herbicide classified as probably carcinogenic to humans, increases TET3 expression and decreases 5mC levels in MCF10A cells. TET3 demethylates the *MT-RNR2-Like Protein 2 (MTRNRL2)* and *Double Homeobox 4 (DUX4)* gene promoters through the binding to their promoters and promotes tumorigenesis in glyphosate-treated MCF10A cells with miR-182-5p over-expression [99]. 

The α-Ketoglutarate Dehydrogenase (KGDH) is an enzyme that catalyzes the oxidative decarboxylation of α-ketoglutarate (α-KG) to succinyl-CoA. Interestingly, (S)-2-[(2,6-dichlorobenzoyl) amino] succinic acid (AA6), an inhibitor of the KGDH enzyme, increases the α-KG and 5hmC levels and decreases the 5mC levels. AA6 decreases the expression of metastasis-associated genes, such as *Zeb1*, inhibiting cell migration and adhesion in CRL2335 and MDA-MB-235 BC cells [100]. 

Finally, Genistein, a bioactive isoflavone present mainly in soybean products, decreases the TET2 and TET3 expression and delays TNBC tumor growth in vivo [101].

## 9. TET Proteins as Biomolecular Tools

Some studies have explored the use of TET proteins to induce the gene-specific demethylation in BC. A fusion protein called TET1CD-dCas9 was created through the CRISPR-Cas9 system with the TET1 Catalytic Domain. This protein promotes site-specific demethylation and an increase in 5hmC levels in the *BRCA1* gene promoter, leading to increased expression in MCF-7 BC cells [102]. Additionally, CRISPR/Cas9 protein fused to TET1 (TET1-dCas9) increases the Forkhead Box P3 (FOXP3) expression via demethylation of their promoters in HCC202 BC cells [103]. Another TET1-dCas9 fusion protein decreases the methylation level in the *Leucine Rich Repeats And Immunoglobulin Like Domains 1 (LRIG1)* promoter, increasing its expression and decreasing the cell viability in HCT116, BT549, MDA-MB-231, and HCC1937 TNBC cells [104]. These results shown that the TET proteins could be useful molecular tools to promote or inhibit the expression of TSG or oncogenes in BC.

## 10. Conclusions

**Figure 3:**
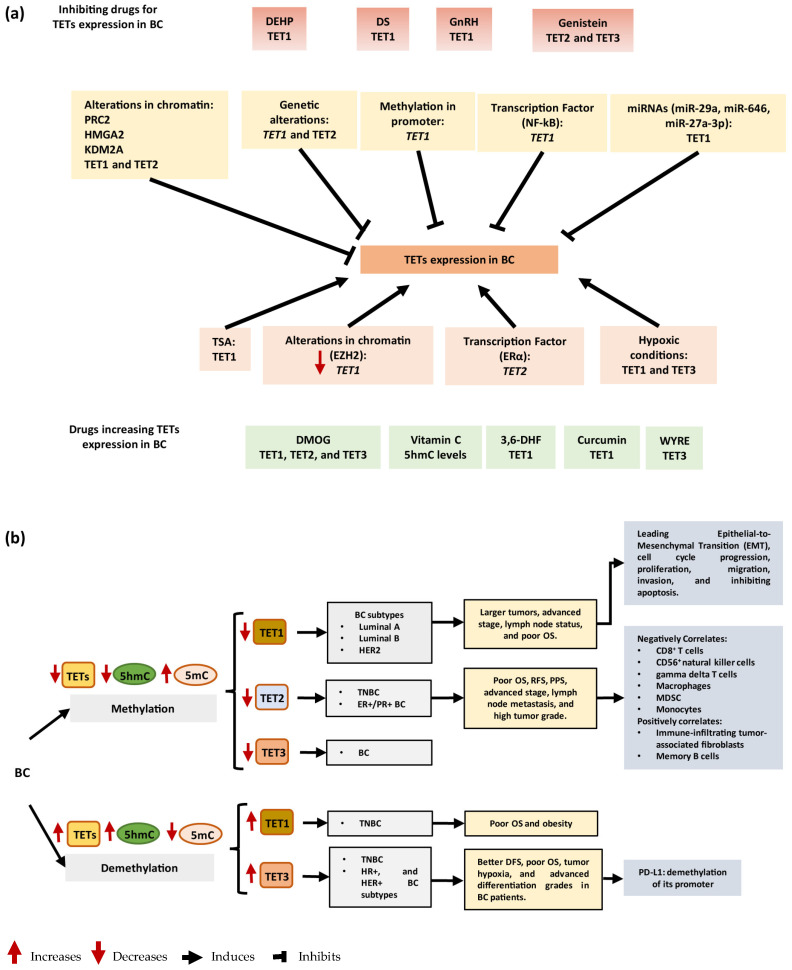
Enzyme TETs in breast cancer. (**a**) Mechanisms of TET deregulation in breast cancer and regulating drug TETs expression. (**b**) Role of TETs in breast cancer. BC = breast cancer; TET = Ten-Eleven Translocation; microRNA = miRNAs; HER2 = Erb-B2 Receptor Tyrosine Kinase 2; DEHP = Di-(2-Ethylhexyl) phthalate; DS = Dioscin; PRC2 = Polycomb Repressive Complex 2; HMGA2 = High Mobility Group AT-Hook 2; KDM2A = Lysine Demethylase 2A; EZH2 = Enhancer Of Zeste 2 Polycomb Repressive Complex 2 Subunit; TSA = Trichostatin A; Erα = Estrogen Receptor 1; OS = Poor Overall Survival; RFS = Relapse-Free Survival; PPS = Post-Progression Survival; DFS = Disease-Free Survival; MDSC = myeloid-derived suppressor cells.

The expression of TET enzymes is dysregulated in BC through several molecules including miRNAs and transcription factors in BC (Figure 3a). The TET dysregulation could serve as a prognostic, diagnostic, and therapeutic biomarker in BC. Furthermore, alterations in the expression or activity of TET enzymes could promote carcinogenesis, progression, and metastasis in patients with BC (Figure 3b). Finally, TET enzymes could be useful tools to induce the specific expression of TSG in BC via genetic engineering. 

## Data Availability

Not applicable.
